# The role of miR‐335‐5p in the redifferentiation of BRAF p.V600E thyroid cancers

**DOI:** 10.1002/1878-0261.70181

**Published:** 2026-06-22

**Authors:** Valeria Pecce, Simone Bini, Marialuisa Sponziello, Giorgio Grani, Giulia Fiscon, Paola Paci, Lorenzo Farina, Rosa Falcone, Valentina Maggisano, Sebastiano Filetti, Cosimo Durante, Antonella Verrienti

**Affiliations:** ^1^ Department of Translational and Precision Medicine Sapienza University of Rome Italy; ^2^ Department for the Promotion of Human Science and Quality of Life San Raffaele Roma University Rome Italy; ^3^ Department of Computer, Control, and Management Engineering “Antonio Ruberti” Sapienza University of Rome Italy; ^4^ Department of Oncology Istituto Dermopatico dell'Immacolata IDI‐IRCCS Rome Italy; ^5^ Department of Health Sciences University “Magna Graecia” of Catanzaro Italy

**Keywords:** computational analysis, miR‐335‐5p, organoids, radioiodine resistance, redifferentiation, thyroid cancer

## Abstract

The BRAF p.V600E mutation activates the RAS/BRAF/MEK/ERK pathway, leading to cancer cell dedifferentiation and uncontrolled growth. In radioiodine‐refractory thyroid cancers, MEK and/or BRAF inhibitors can induce redifferentiation, resensitizing tumors to radioiodine. However, compensatory mechanisms limit this efficacy. We used the SWitchMiner software to identify a small pool of regulatory genes, called switch genes, critically associated with drastic changes in cell phenotypes, using TCGA transcriptomic data from *BRAF*‐mutant papillary thyroid carcinoma and normal thyroid tissues, which highlighted miR‐335‐5p. Restoring miR‐335‐5p in thyroid cancer cell lines harboring the *BRAF* mutation increased expression of thyroid‐specific genes and proteins, especially in well‐differentiated cell lines, with enhanced sodium‐iodide symporter localization and iodine uptake confirmed in organoids. Due to the connection between thyroid‐specific and EMT‐related genes in the protein–protein interaction network, we examined how miR‐335‐5p overexpression affects EMT pathway genes that modulate thyroid‐specific genes and Kinase Inhibitor (KI) resistance. miR‐335‐5p inhibited the expression of nearly all analyzed genes in less‐differentiated thyroid cell lines. Thus, miR‐335‐5p may be a viable therapeutic target to restore radioiodine avidity in *BRAF*‐mutant metastatic thyroid cancer and enhance KI treatment redifferentiation.

## Graphical abstract

The BRAF p.V600E mutation promotes thyroid cancer dedifferentiation and radioiodine resistance. Using a *network* approach, we identified miR‐335‐5p as a key regulator of BRAF‐mutated thyroid tumors. Restoring miR‐335‐5p increased thyroid‐specific gene expression and iodine uptake in cells and organoids. It also inhibited EMT‐related genes associated with KI resistance. Therefore, miR‐335‐5p could enhance redifferentiation and radioiodine sensitivity in BRAF‐mutant thyroid cancer.

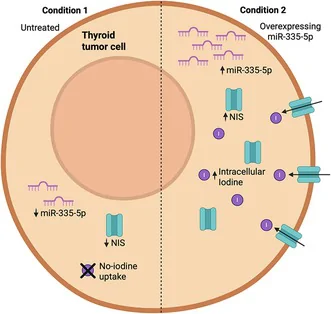

**Abbreviations**
ATC: anaplastic thyroid carcinoma
BRAF: B‐Raf proto‐oncogene
BRAFi: BRAF inhibitor
CDH1: E‐cadherin
CDH11: Cadherin 11
CDH16: kidney‐specific cadherin
CDH2: N‐cadherin
DEGs: differentially expressed genes
DHGTC: differentiated high‐grade thyroid carcinoma
DTC: differentiated thyroid carcinoma
EGFR: epidermal growth factor receptor
EMT: epithelial‐mesenchymal transition
FN1: fibronectin 1
FTC: follicular thyroid cancer
I‐131: iodine‐131
IEFVPTC: invasive encapsulated follicular variant papillary carcinoma
KI: kinase inhibitor
LATS2: large tumor suppressor kinase 2
MAPK: mitogen‐activated protein kinase
MEKi: MEK inhibitor
MET: MET proto‐oncogene
miRNA: microRNA
MMP9: matrix metalloproteinase 9
NIS: sodium‐iodide symporter
OCA: oncocytic carcinoma
PAX8: paired box 8
PDE5A: phosphodiesterase 5A
PDTC: poorly differentiated thyroid carcinoma
PI3K: phosphatidylinositol 3‐kinase
PLAUR: plasminogen activator, urokinase receptor
PPi/PPI: protein–protein interaction
PTC: papillary thyroid carcinoma
RAI: radioiodine
RAIR: radioiodine‐refractory
RUNX2: runt‐related transcription factor 2
SWIMmeR: SWitchMiner
TAZ: transcriptional coactivator with PDZ‐binding motif
TCF3: transcription factor 3
TCGA: The Cancer Genome Atlas
TG: thyroglobulin
TPO: thyroid peroxidase
TRβ: thyroid hormone receptor beta
TSHR: thyroid‐stimulating hormone receptor
YAP1: yes associated protein 1

## 1 Introduction

The majority of thyroid tumors originate from thyroid follicular cells. Malignant neoplasms derived from follicular cells are divided into well‐differentiated thyroid cancers, including papillary carcinoma (PTC), invasive encapsulated follicular variant papillary carcinoma (IEFVPTC), follicular thyroid cancer (FTC), and oncocytic carcinoma (OCA); high‐grade thyroid cancers, that is, differentiated high‐grade carcinoma (DHGTC) and poorly differentiated carcinoma (PDTC); and anaplastic carcinoma (ATC) [1]. Less‐differentiated thyroid cancers, specifically PDTC and ATC, may develop *de novo* or result from the stepwise dedifferentiation of papillary and follicular carcinomas [1].

The mutations involved in thyroid carcinogenesis are those found in the effectors of the mitogen‐activated protein kinase (MAPK) pathway and the phosphatidylinositol 3‐kinase (PI3K)–AKT pathway, crucial for tumor initiation and progression, respectively. The mutated genes that affect these pathways encode cell membrane receptor tyrosine kinases *RET* and *NTRK1* and intracellular signal transducers *BRAF* and *RAS* [2]. Among the mutations of *MAPK*s, the most frequent, harbored by 60–70% of PTC, is the p.V600E mutation of the *BRAF* gene that has been associated with reduced expression of key genes involved in iodine metabolism (iodide uptake, thyroid hormone synthesis, and differentiation, i.e., *NIS*; *TPO*; *TG*; *PAX8*) [3]. The consequence is that these cancers lose the ability to concentrate iodine efficiently, rendering radioiodine (RAI, iodine‐131; I‐131) treatment ineffective [4]. RAI refractory (RAIR) thyroid cancer has a dismal prognosis, with an estimated 10‐year survival rate of 10% [5]. Management options in this subset of patients include active surveillance, local treatments for metastatic sites, or kinase inhibitor (KI) therapy for rapidly progressing, symptomatic, or life‐threatening diseases [6, 7].

A novel strategy called *redifferentiation* is being explored to restore the capacity of RAIR tumors to trap and respond to a minimal administration of I‐131 [8]. This strategy consists of administering KIs targeting critical molecules involved in NIS regulation (MEK and/or BRAF inhibitors) for a period necessary to induce redifferentiation in RAIR thyroid cancer, thus resensitizing tumors to RAI, followed by therapeutic radioiodine [7].

However, treatment with kinase inhibitors presents several significant issues, including the emergence of both primary and acquired resistance to these drugs and chronic and harmful adverse effects [9]. These limits make the discovery of new drugs necessary.

Both genetic and epigenetic factors are known to regulate the expression of thyroid‐specific genes [10]. Given their role in cellular epigenetic regulation, microRNAs might be an alternative approach for redifferentiating RAIR thyroid cancer [11]. Indeed, the refractoriness of thyroid carcinomas to radioiodine has been associated with the deregulation of microRNAs, particularly the downregulation of miR‐139‐5p, in RAIR carcinomas compared to RAI responder ones [12].

The molecular mechanisms behind redifferentiation are complex and only partially understood.

A deeper understanding of the molecular process implicated in the pathogenesis and de/redifferentiation of thyroid cancer will enable the identification of more effective therapeutic targets.

A powerful emerging approach is the study of cancer molecular landscapes using bioinformatic tools to identify hidden, tissue‐specific pathways, predict the effectiveness of existing therapeutic agents (drug repurposing), or suggest new ones. SWitchMiner (SWIMmeR) is software developed in R that can identify a small pool of regulatory genes, called switch genes, critically associated with drastic changes in cell phenotypes [13]. This tool has been successfully applied in human cancers such as breast cancer [14, 15] and glioblastoma [16].

In previous work, we used SWIM software to analyze RNA‐sequencing data from 249 thyroid cancer samples with the *BRAF* p.V600E mutation and their normal tissues from The Cancer Genome Atlas cohort (TCGA; https://cancergenome.nih.gov/). This analysis identified 227 switch genes from 2167 differentially expressed genes (DEGs), including protein‐coding genes, long noncoding RNAs, and pseudogenes [17].

In this study, we further analyzed the 227 switch genes using a different *in silico* approach to understand the mechanisms involved in thyroid tumorigenesis driven by the p.V600E mutation of the *BRAF* gene. We identified miRNA‐335‐5p as the main regulator of most of those genes. Using immortalized, primary, and 3D‐cultured thyroid cell lines, we provided *in vitro* evidence of this miRNA's role in thyroid cancer dedifferentiation and progression.

## 2 Materials and methods

### 2.1 Computational analysis

We applied SWitchMiner (SWIM) [13], a well‐established network‐based methodology that integrates differentially expressed genes within a co‐expression network framework to predict key genes affected by the disease of interest and to identify correlated molecular patterns. By considering the topological properties of the nodes and assessing their functional roles according to their ability to convey information within and across network modules, SWIM identifies a small subset of genes, known as switch genes, which display peculiar topological features within the gene co‐expression network: (a) They exhibit coherent correlation patterns, suggesting potential coregulation or functional relationships; (b) they are not local hubs in their modules but rather act as connectors across clusters, thus enabling information flow between modules; and (c) they are mainly anticorrelated with their interaction partners. Switch genes have been shown to play a crucial role in phenotype transitions (e.g., from physiological to pathological states) and have been successfully applied in various biological contexts, ranging from plants (*Vitis vinifera*) [18] to human diseases, including several cancers [16, 19], with experimental validation in glioblastoma stem cells [20]. SWIM, originally developed in matlab [13], is also available as an open‐source R implementation (SWIMmeR) [21]. Starting from the switch genes previously identified and reported in Table S1 [22], we performed a microRNA‐target enrichment analysis using the MicroRNA ENrichment TURned NETwork (MIENTURNET) tool [23] (http://userver.bio.uniroma1.it/apps/mienturnet/), which integrates both computationally predicted and experimentally validated miRNA‐target interactions from TargetScan and miRTarBase databases, respectively. Only experimentally validated interactions were considered in this study. Final miRNA selection was based on those with the highest number of experimentally validated targets and with a false discovery rate (FDR) < 0.05. In parallel, we constructed the protein–protein interaction (PPI) network among switch genes using the STRING platform and analyzed its cluster structure through the density‐based local search clustering algorithm DBSCAN.

### 2.2 Cell cultures and organoids

Primary cell cultures were established, as previously described in Dima et al., from the fresh specimens of patients with benign tumors (i.e., follicular thyroid adenomas) and papillary thyroid cancer who underwent total thyroidectomy [24]. The generated cell lines were maintained in culture form 2‐5 passages at 37 °C with 5% of CO_2_. The presence of p.V600E point mutation of the *BRAF* gene in the cell lines was verified by Sanger sequencing analysis of DNA isolated using NucleoSpin Tissue Mini kit for DNA from cells and tissue (Macherey Nagel, Nordrhein‑Westfalen, Germany) as previously described [25].

Commercial immortalized cell lines [NTHYORI (CVCL_2659), TPC1 (CVCL_6298), BCPAP (CVCL_0153), K1 (CVCL_2537), 8505c (CVCL_1054), SW1736 (CVCL_3883), FTC‐133 (CVCL_1219)] were cultured in DMEM or RPMI medium (Gibco‐BRL Division, Thermo Fisher Scientific, Waltham, MA, USA) according to ATCC^®^ instructions, containing 10% FBS (Gibco‐BRL Division, Thermo Fisher Scientific) and Antibiotic–Antimycotic solution (Gibco‐BRL Division, Thermo Fisher Scientific). All cell lines were provided by ATCC or ECACC‐Cell‐Lines from Merck, Darmstadt, Hesse, Germany. All cell lines have been authenticated in the past 3 years. The mutational status of each immortalized cell line was verified by Sanger sequencing analysis to confirm the mutational status previously reported [26]. All cell lines were monitored for mycoplasma infection every 6 months to assess the work with mycoplasma‐free cells. Organoids were established from both primary cells and cultured using the conditioned medium, as Pecce et al. reported in 2022 [27].

### 2.3 Cell treatments and proliferation assay

Synthetic miR‐335‐5p (MISSION^®^ microRNA Mimic hsa‐miR‐335‐5p HMI0490) or negative control (MISSION^®^ miRNA, Negative Control 1, HMC0002) were transfected into primary and immortalized thyroid cancer cell lines at 30 nm of final concentration using Lipofectamine 3000 Transfection Reagent (Thermo Fisher Scientific). Treatments, according to manufacturer instructions, were performed after 2 h of cell starvation and maintained for 48 h. Cell viability before and after treatments was measured using the WST‐1 assay (Sigma‐Aldrich division, Merck, NJ, USA) after 24 and 48 h, according to the manufacturer's instructions. The viability was monitored for 5 h during the WST‐1 induction.

### 2.4 Cells RNA isolation and quantitative RT‐PCR

RNA was isolated from cells using the RNeasy Mini Kit (Qiagen, Hilden, Germany) and quantified with the NanoDrop 2000 (Thermo Fisher Scientific). The High‐Capacity cDNA Reverse Transcription Kit (Applied Biosystems Life Technologies, Thermo Fisher) was used to synthesize cDNA from RNA according to the manufacturer's instructions (Applied Biosystems, Carlsbad, CA, USA). The expression levels of miR‐335‐5p, thyroid‐specific genes (*NIS*, *TPO*, *TG*, *PAX8*, *TSHR*), and EMT‐related genes (*FN1*, *MET*, *RUNX2*, *PDE5A*, *PLAUR*, *TCF3*, *LATS*, *TAZ*, *YAP1*, *CDH11*, *CDH1*, *CDH2*, *CDH11*, *MMP9*) were analyzed on cDNAs from treated and control cells using TaqMan gene expression Assay‐on‐Demand and TaqMan Universal Master Mix according to the manufacturer's instructions (Thermo Fisher Scientific).

Results were calculated using the 2^−ΔΔCt^ method and normalized to the corresponding endogenous control samples, U6 snRNA (TaqMan^®^ Gene Expression Assays Code: 001093), *GAPDH* (TaqMan^®^ Gene Expression Assays Hs99999905_m1), or *B2M* (Hs99999907_m1). Data were expressed as mean + SD of three replicates [28].

The TaqMan^®^ Gene Expression Assays used are *SLC5A5* (Hs00166567_m1), *TG* (Hs00174974_m1), *PAX8* (Hs00247586_m1), *TPO* (Hs00892519_m1), *TSHR* (Hs01053846_m1), miR‐335‐5p (Code: 478324_mir), *FN1* (Hs00365052_m1), *MET* (Hs01565584_m1), *RUNX2* (Hs00231692_m1), *PDE5A* (Hs00153649_m1), *PLAUR* (Hs00958880_m1), *TCF3* (Hs00413032_m1), *LATS* (Hs01125528_m1), *TAZ* (Hs01125528_m1), *YAP1* (Hs00902712_g1), *CDH11* (Hs00901475_m1), *CDH1* (Hs01023894_m1), *CDH2* (Hs00983056_m1), *CDH11* (Hs00901475_m1), *MMP9A* (Hs00234579_m1).

### 2.5 Immunofluorescence

Immunofluorescence experiments were performed using Lab‐Tek chamber slides as support. Cells were fixed with 4% paraformaldehyde for 20 min at room temperature, permeabilized with Triton X‐100 (0.1%) diluted in phosphate‐buffered saline (PBS), and incubated in blocking solution (BSA 3% in PBS). Cells were incubated overnight with primary antibodies diluted in PBS and for 1 h with secondary antibodies. Primary antibodies were anti‐TPO (Abcam, Cambridgeshire, UK), anti‐TSHR (Abcam), anti‐PAX8 (Cell Signaling, Danvers, MA, USA), and anti‐NIS (Genetek Biopharma GmbH, Berlin, Germany) diluted 1 : 50. Secondary antibodies (488‐conjugated anti‐rabbit) were purchased from Thermo Fisher Scientific. Nuclei were Hoechst‐counterstained. Images were acquired with an optical microscope, Leica DM IL LED Fluo, using a GFP filter cube.

### 2.6 Invasion assay

Cell invasion was evaluated using Transwell chambers with 8‐μm pore membranes (Corning Inc., Corning, NY, USA). For invasion assays, the upper surface of the membranes was coated with a 50 μL layer of extracellular matrix gel (Matrigel, Corning) and allowed to solidify for 30–60 min at 37 °C. Control cells and cells treated with miR‐335‐5p for 48 h were harvested, resuspended in serum‐free medium, and then seeded into the upper chambers, while the lower chambers were filled with complete medium containing 10% FBS as a chemoattractant. After 24 and 48 h of incubation, noninvaded cells were removed from the upper surface of the membranes using a cotton swab. Cells that had invaded the underside of the membranes were fixed in 70% of methanol for 10 min and stained with 0.1% crystal violet at room temperature. Inserts were then washed thoroughly with distilled water and air‐dried. Stained cells were visualized under an inverted microscope, and three random fields per membrane were counted to quantify invasion.

### 2.7 Iodide uptake assay

Iodide uptake assay was performed as previously described [29]. Briefly, cells were seeded in a 96‐well plate at 70% of confluence and, the day after, were treated as described in the cell treatments section. After 48 h of treatments, iodide uptake assay was performed.

The culture medium was replaced twice by the uptake buffer (containing 10 mm of Hepes diluted in Hank's balanced salt solution [HBSS]), and at the end of the washing cycle, 80 μL of fresh uptake buffer remained in each well of a 96‐well plate. Immediately, 10 μL of 100 μm NaI solution was added to each well. For each experimental condition, a competitive inhibitor of NIS (KClO_4_ 10 μm) was added in half of the wells as a control to determine the nonspecific iodide uptake. The assay plate was left at 20 °C for 60 min in the dark. At the end of incubation, the buffer was discarded, and cells were washed once with ice‐cold HBSS (Thermo Fisher Scientific). Then, cells were lysed with 100 μL of a 0.01 m NaOH solution. The whole lysate was used for the Nonradioactive Iodide Assay kit (Bertin Pharma, Montigny‐le‐Bretonneux, Île‐de‐France, France) according to the manufacturer's instructions. The iodide concentration in unknown samples was determined using linear regression of the standard curve provided by the kit. Results were expressed as specific units (μm) of iodide accumulation relative to control.

### 2.8 Western blot analysis

Cells were lysed using the buffer described by Pecce et al. [30]. About 30 μg of total protein extracts was loaded on 12% polyacrylamide gel, transferred to PVDF membranes, blocked with 5% nonfat dry milk for 2 h, and then incubated overnight with primary antibodies.

The primary antibodies were anti‐TPO (Abcam), anti‐TSHR (Abcam), anti‐PAX8 (Cell Signaling), anti‐NIS (Genetek) diluted 1 : 1000, and anti‐βACTIN (Sigma‐Aldrich) diluted 1 : 3000, used as a loading control. The membranes were then incubated with horseradish peroxidase‐conjugated secondary antibodies: anti‐Rabbit (diluted 1 : 5000) or anti‐mouse (diluted 1 : 5000) (Transduction Laboratories, Lexington, KY, USA). The blots were developed with the western blot ECL Plus detection system (Perkin Elmer, Milan, Italy), and the results were acquired with the ChemiDoc MP system (Bio‐Rad) and analyzed with image lab software (Bio‐Rad).

## 3 Results

### 3.1 miR‐335‐5p targets most of the switch genes that characterize the BRAF p.V600E thyroid cancer network

This study builds upon a prior analysis conducted using SWIM software, published by our group in 2019 [22]. That previous paper investigated RNA‐sequencing data from 294 thyroid cancer samples harboring the *BRAF* p.V600E mutation, along with their corresponding normal tissues (*n* = 59) from the TCGA cohort. As detailed in Table S1, the analysis identified 227 switch genes among 2167 differentially expressed genes (DEGs), which include protein‐coding genes, long noncoding RNAs, and pseudogenes [17].

To identify the mechanisms that characterize the tumorigenesis in *BRAF*‐mutated thyroid cancer, we used all 227 switch genes as input for miRNA‐target enrichment analysis via the MIENTURNET platform, selecting only the experimentally validated interactions. We identified miR‐335‐5p based on the number of experimentally validated targets and the false discovery rate (FDR) value (Fig. 1C). Notably, approximately 30% (63 out of 227) of the switch genes were classified as experimentally validated targets of miR‐335‐5p; the list is detailed in Table S2.

**Fig. 1:**
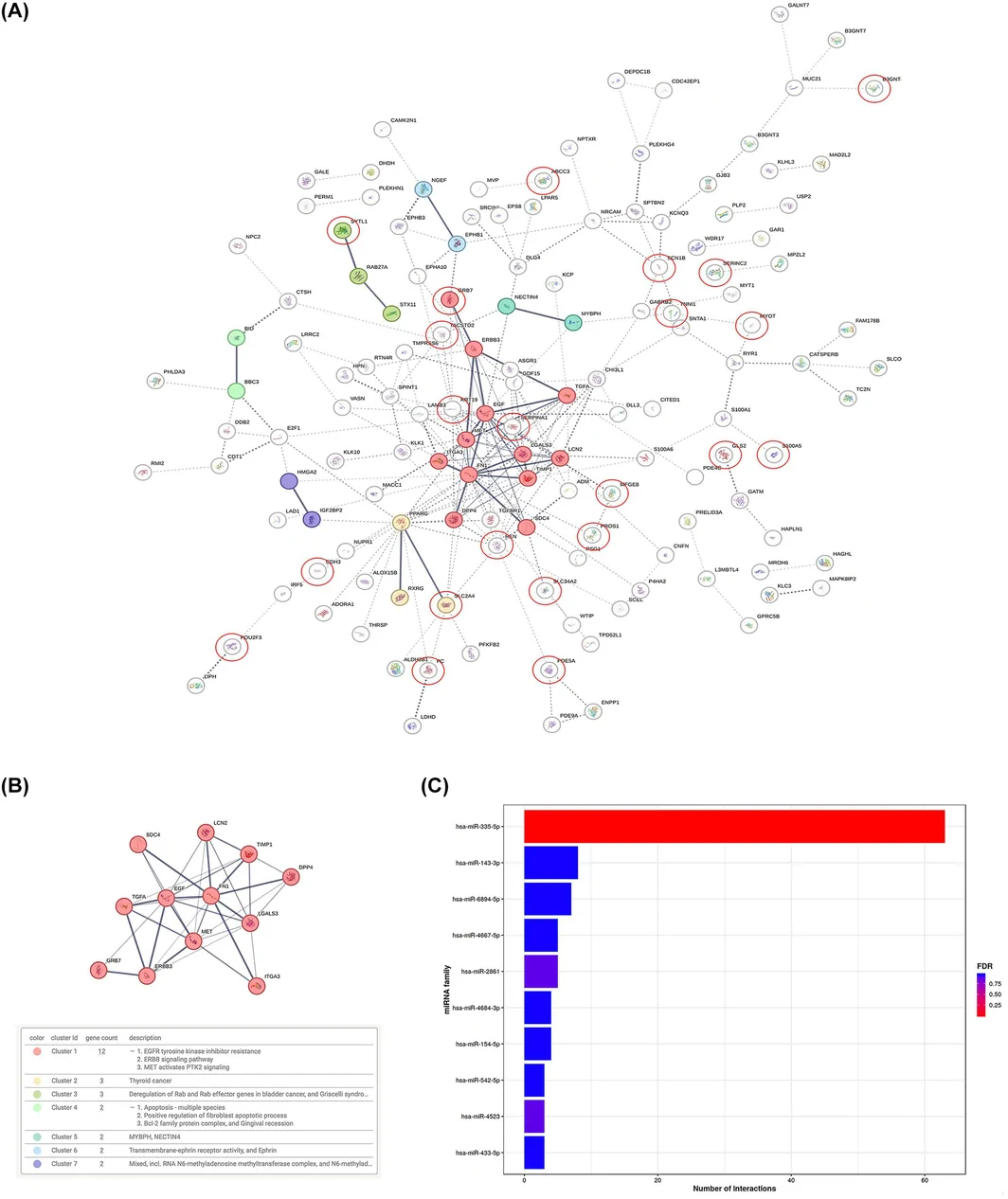
Analysis of switch genes involved in *BRAF*‐mediated thyroid carcinogenesis. (A) Graphical representation of the protein–protein Interaction (PPI) network of connected switch genes built using the Search Tool for the Retrieval of Interacting Genes (STRING) (interactive PPI available at https://version‐12‐0.string‐db.org/cgi/network?networkId=bHdszLxLTpOB), the 22 out of 63 targets of miR‐335‐5p (red circles) that are part of the BRAF thyroid cancer network, in red (cluster 1), yellow (cluster 2), olive green (cluster 3), seafoam green (cluster 4), green (cluster 5), light blue (cluster 6), and purple (cluster 7). (B) The table shows the seven clusters identified by STRING enrichment analysis and reported in the PPI network in panel (A). The largest cluster, cluster 1, is plotted. (C) miRNA‐target enrichment analysis of switch genes performed using MIcroRNA ENrichment TURned NETwork (MIENTURNET) software. The false discovery rate (FDR) is reported from blue (> 0.75) to red (< 0.25).

To better understand the relationship among the switch genes that characterize the *BRAF*‐mutated thyroid cancer, we analyzed the protein–protein interaction (PPi) Network of the 227 switch genes using the STRING platform, widely used for hypothesis generation, to highlight and visualize their potential interactions and interconnections (Fig. 1A). In the network, seven clusters are identifiable. The largest cluster contains genes involved in the EGFR tyrosine kinase inhibitor resistance, ERBB, and MET/PTK2 pathways (including 12 switch genes) (Fig. 1B). These genes are implicated in the epithelial‐mesenchymal transition (EMT), a physiological process that occurs during tumor growth and cancer progression, allowing epithelial cells to acquire the migratory and invasive characteristics of mesenchymal cells [26]. The second largest cluster relates to *thyroid cancer* and includes three switch genes. The interactive PPi network is available at the following link https://version‐12‐0.string‐db.org/cgi/network?networkId=bHdszLxLTpOB.

Interestingly, the pathway‐enrichment analysis of the 63 miR‐335‐5p targets did not identify any significant clustering, with the miR‐335‐5p target genes scattered throughout the network, as shown in Fig. 1A (red circles). This finding suggests that the switch genes targeted by miR‐335‐5p have a variety of functions not belonging to a specific pathway, highlighting that miR‐335‐5p may play a pivotal role in *BRAF* p.V600E‐driven thyroid carcinogenesis by modulating multiple functions.

### 3.2 miR‐335‐5p is downregulated in thyroid cancer cell lines

To investigate the putative role of miR‐335‐5p in the *BRAF* p.V600E‐driven thyroid carcinogenesis, we selected normal, benign, and malignant thyroid cell lines with different degrees of dedifferentiation to assess the miR‐335‐5p expression level differences.

As shown in Fig. 2A, we assessed miR‐335‐5p expression levels in primary cell lines derived from two benign tumors (i.e., thyroid follicular adenomas) and three papillary thyroid carcinomas (PTCs), with the latter harboring the p.V600E mutation of the *BRAF* gene. As a control, we used cell lines from the normal thyroid counterparts of the three patients with PTC.

**Fig. 2:**
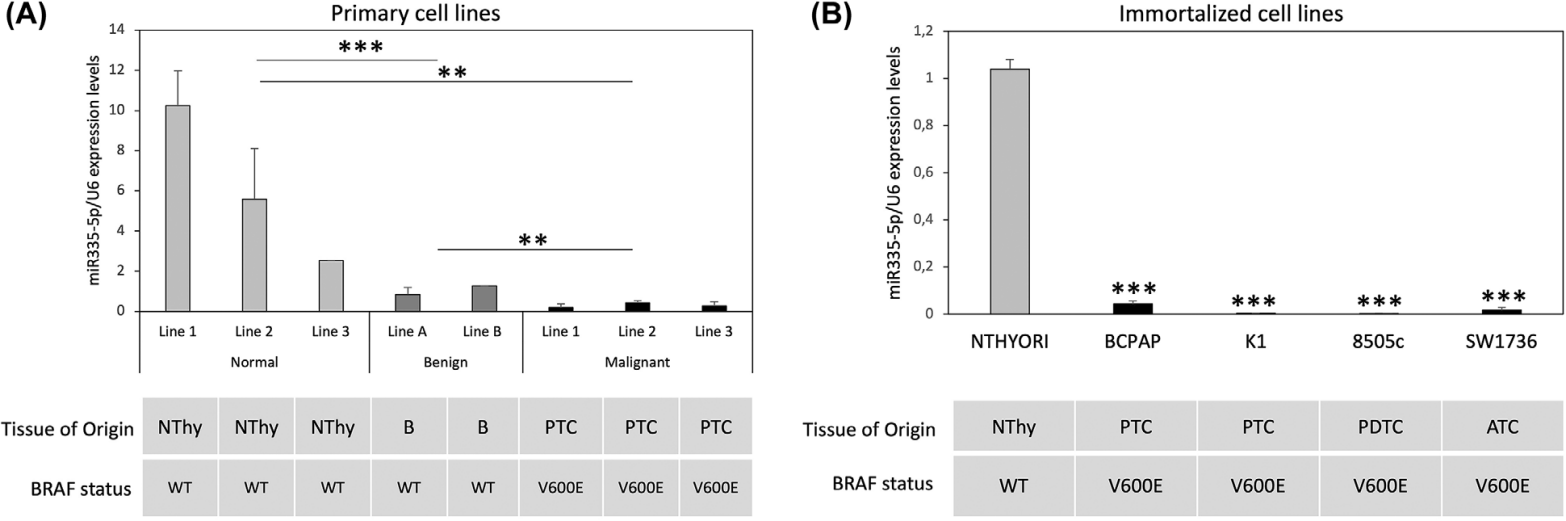
Expression levels of miR‐335‐5p in thyroid cell lines. (A) Relative expression levels of miR‐335‐5p in primary normal thyroid cell lines 1, 2, and 3, benign thyroid tumor lines A and B, and thyroid cancer cell lines 1, 2, and 3. (B) Relative expression levels of miR‐335‐5p in 4 immortalized thyroid cancer cell lines with p.V600E mutation of the *BRAF* gene. Data from three independent biological replicates (*N* = 3) are expressed as 2^−ΔΔCt^ value (mean ± SD), normalized to U6 small nuclear RNA (snRNA), and compared with the normal cell lines NTHYORI in both graphs. *P* value < 0.05, *; 0.005 **; 0.0005 *** (*t*‐test data in technical replicates). ATC, anaplastic thyroid cancer; B, benign; NThy, normal; PDTC, poorly differentiated thyroid cancer; PTC, papillary thyroid cancer.

The analysis revealed a consistent downregulation of miR‐335‐5p in all primary tumor cell lines compared to normal cells, with a moderate decrease in benign lines and an even greater reduction in malignant lines.

We also evaluated miR‐335‐5p levels in four immortalized thyroid cancer cell lines harboring the same *BRAF* p.V600E mutation to add evidence to these findings. The cell lines selected have various degrees of differentiation, including well‐differentiated (BCPAP, K1), poorly differentiated (8505c), and anaplastic (SW1736) thyroid cancers [26]. The immortalized normal thyroid cell line NTHYORI was used as the control. As shown in Fig. 2B, all immortalized cancer cell lines exhibited markedly lower miR‐335‐5p expression than the control cell line, regardless of their differentiation status.

Lastly, we used TPC1 and FTC‐133 as wild‐type *BRAF* (*BRAF*‐wt) control cell lines, which harbor the driver mutations *CCDC6::RET* rearrangement and *PTEN* (p.R130*), respectively. As shown in Fig. S1A, a nonstatistically significant decrease in the level of microRNA‐335‐5p was observed in the TPC1 and FTC‐133 cell lines compared with the NTHYORI control cells. Our findings highlight the consistent downregulation of miR‐335‐5p only in *BRAF* p.V600E‐mutant thyroid cancer cells, suggesting its potential involvement in the *BRAF* p.V600E‐driven carcinogenesis and progression.

### 3.3 The overexpression of miR‐335‐5p increases the expression of thyroid‐specific markers

To investigate the potential role of miR‐335‐5p in the *BRAF* p.V600E‐driven thyroid cancers, we restored miR‐335‐5p expression in six thyroid cancer cell lines: primary cell lines 1 and 2, BCPAP, K1, 8505c, and SW1736. As shown in Fig. S2, miR‐335‐5p overexpression was successfully achieved in all six cell lines after 48 h of miRNA transfection, as well as in the cell lines used as control, the NTHYORI, and in the cell lines used as *BRAF*‐wt control ones (Fig. S1B). To assess the viability of all cell lines used in miR‐335‐5p overexpression conditions, we performed the WST‐1 assay both before and after miR‐335‐5p treatment, monitoring viability at 24 and 48 h. As shown in Fig. 3, a minimal, nonstatistically significant difference was observed in all cell lines after 24 h of miR‐335‐5p overexpression. After 48 h of treatment, the 8505c and SW1736 cell lines exhibited a significant reduction in viability, maintaining a growth trend similar to that observed in the control cells.

**Fig. 3:**
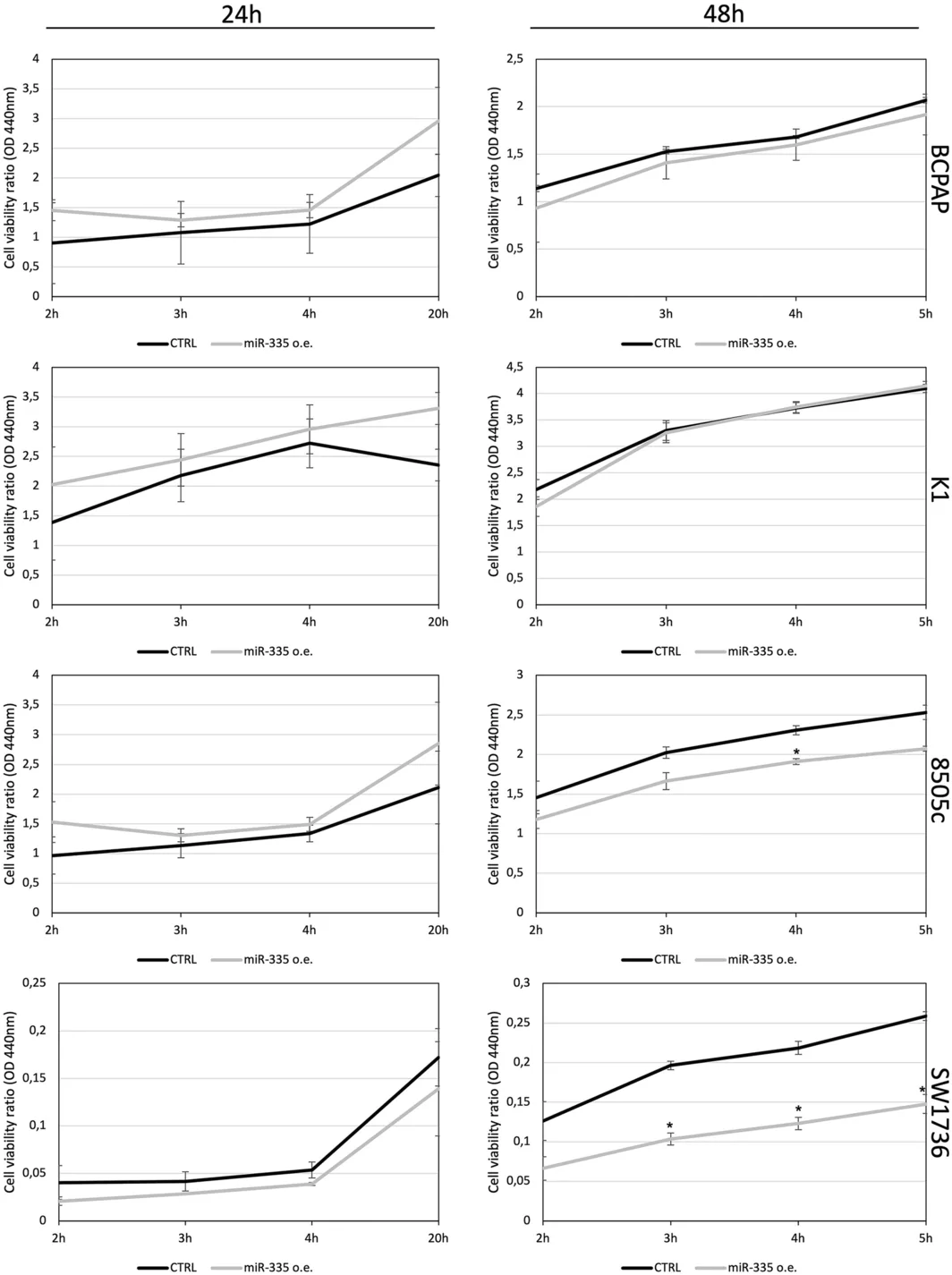
Viability quantification after miR‐335‐5p overexpression. Viability quantification using the WST‐1 assay. Data represent the optical density (OD) values at 440 nm of *BRAF*‐mutated cell lines after 24 h and 48 h of miR‐335‐5p overexpression. Each panel displays the control and treatment curves, expressed as mean ± SD, with *P* values of < 0.05 (*), < 0.005 (**), and < 0.0005 (***) (*t*‐test data from technical replicates). The experiment was repeated three times (*N* = 3).

Moreover, to analyze its role in the differentiation process, we analyzed the expression levels of thyroid‐specific genes (*NIS*, *TPO*, *TSHR*, *PAX8*, and *TG*) before and after overexpression of miR‐335‐5p. Figure 4A shows that miR‐335‐5p overexpression led to increased expression of *NIS*, *TPO*, *TSHR*, and *PAX8* transcripts in both primary lines. Notably, the increase was significant for *NIS*, *TSHR*, and *PAX8* in primary cell line 1 and for *TPO* and *TSHR* in primary cell line 2. Similarly, two of the four immortalized cell lines, specifically the most differentiated among the *BRAF*‐mutated lines selected, BCPAP and K1, exhibited significantly elevated levels of all five thyroid‐specific transcripts (i.e., *NIS*, *TPO*, *TSHR*, *PAX8*, and *TG*) after miR‐335‐5p restoration (Fig. 4B). Regarding control cell lines, the normal lines showed a slight increase in expression levels for TPO and NIS. In contrast, the *BRAF*‐wt lines, TPC1 and FTC‐133, exhibited lower levels and no changes in thyroid‐specific genes after overexpression of miR‐335‐5p (Fig. S1C).

**Fig. 4:**
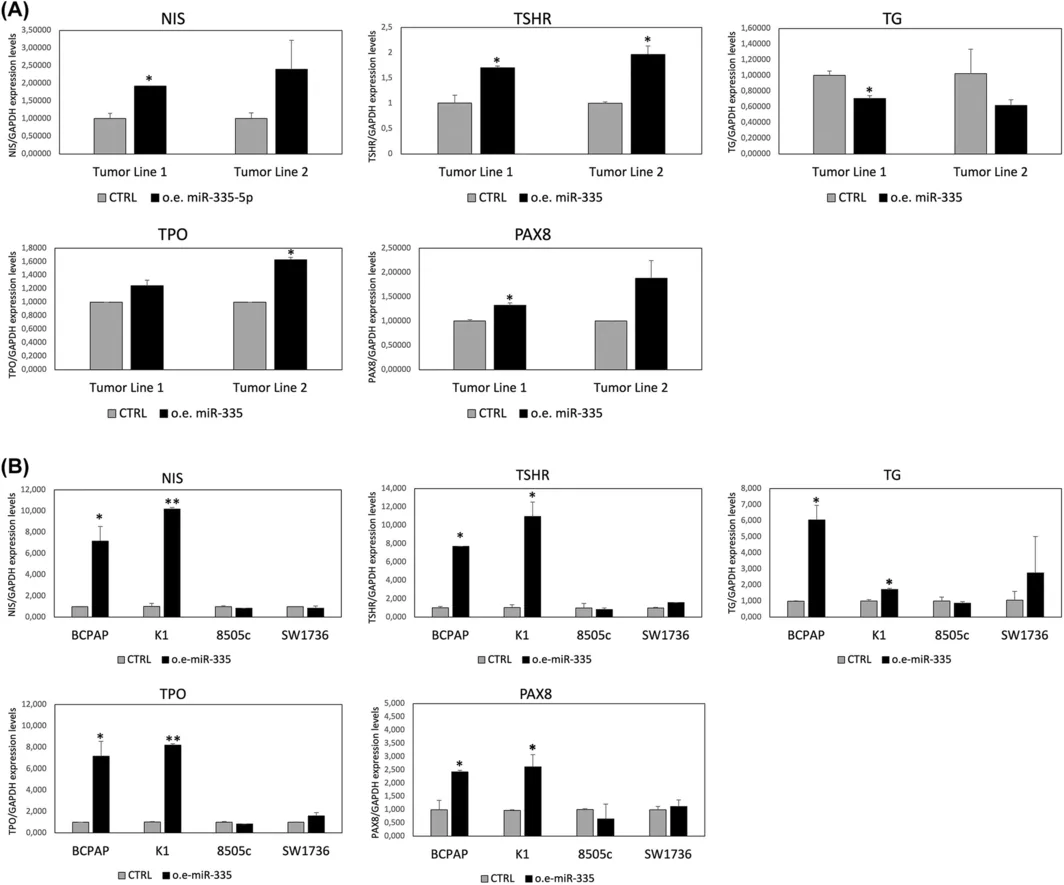
Thyroid‐specific gene expression in thyroid cancer cell lines. *NIS*, *TSHR*, *TG*, *TPO*, and *PAX8* expression levels in primary (A) and immortalized (B) thyroid cancer cell lines 48 h after miR‐335‐5p overexpression. Data from three independent biological replicates (*N* = 3) are expressed as 2^−ΔΔCt^ value (mean ± SD), normalized to the endogenous control (GAPDH), and compared with control cells (CTRL). *P* value < 0.05*; 0.005**; 0.0005*** (*t*‐test data).

To further explore the molecular changes influencing thyroid cell differentiation in response to miR‐335‐5p, we measured total TPO, TSHR, NIS, and PAX8 protein levels in the four immortalized cell lines. Consistent with the transcript data, we observed increased protein expression in the K1 cell line following overexpression of miR‐335‐5p. Additionally, BCPAP cells showed a significant increase in NIS protein levels. In contrast, no significant changes in thyroid‐specific protein expression were detected in the two less‐differentiated cell lines, 8505c and SW1736 (Fig. 5).

**Fig. 5:**
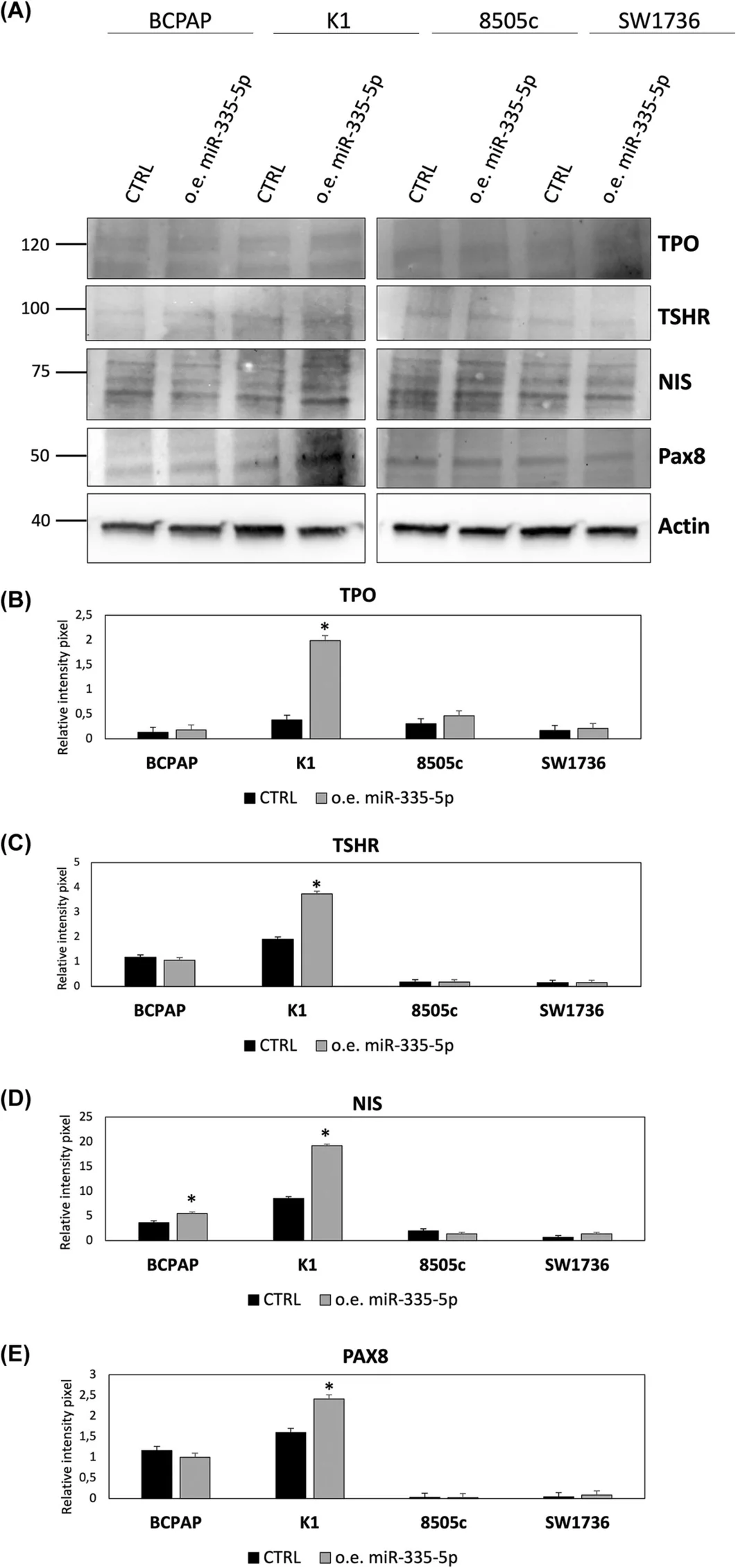
Thyroid‐specific protein expression in immortalized cell lines. (A) Representative western blot of thyroid‐specific protein in immortalized cell lines after 48 h of overexpression of miR‐335‐5p; β‐actin was used as a loading control. Densitometric analyses from three independent biological replicates (*N* = 3) are shown as the mean ± SD for TPO (B), TSHR (C), NIS (D), and PAX8 (E). *P* value < 0.05*; 0.005**; 0.0005*** (*t*‐test data).

In the normal and *BRAF*‐wt control cell lines (NTHYORI, TPC1, and FTC‐133), unlike the cell lines that harbor the *BRAF* p.V600E mutation, miR‐335‐5p overexpression did not lead to increased expression of thyroid‐specific proteins, as detailed in Fig. S1 (panels D and E).

These findings suggest that restoring miR‐335‐5p levels induces redifferentiation in *BRAF* p.V600E mutant thyroid cells, with different responses depending on the cell lines' differentiation rates.

### 3.4 miR‐335‐5p overexpression enhances the expression and subcellular localization of thyroid‐specific markers and reactivates NIS in thyroid cancer cells

To better understand miR‐335‐5p's role in regulating thyroid‐specific markers, we investigated the subcellular localization of thyroid‐specific proteins.

As shown in Fig. 6, we analyzed the subcellular localization of NIS and TSHR in the four immortalized thyroid cancer cell lines after 48 h of miR‐335‐5p overexpression. This analysis revealed an increase in the expression and colocalization of NIS and TSHR at the cell membrane, especially in the K1 cell line.

**Fig. 6:**
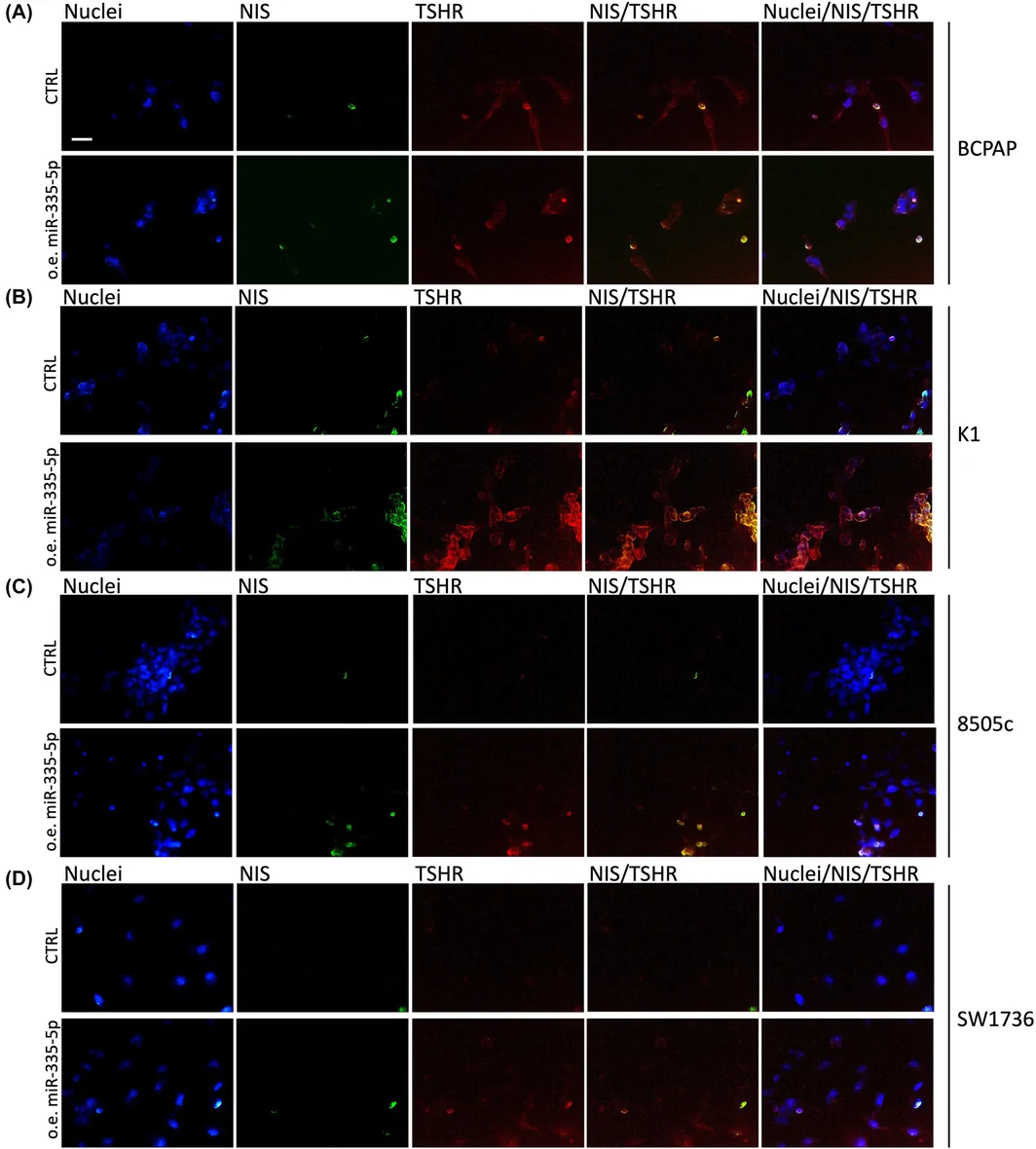
NIS and TSHR localization in immortalized cell lines. Immunofluorescence of NIS and TSHR in BCPAP (A), K1 (B), 8505c (C), and SW1736 (D) cell lines before and after 48 h of miR‐335‐5p overexpression. The blue stain represents nuclei (Hoechst), the green stain represents NIS, and the red stain represents TSHR. The images are represented at 40× magnification, with a scale bar of 100 μm. Representative immunofluorescence of three biologically independent replicates (*N* = 3).

To further explore whether miR‐335‐5p overexpression enhances cancer cell redifferentiation, we generated organoids using primary cancer cell lines 1 and 2. These organoids, cultured in an extracellular matrix with a conditioned medium, as described by Pecce in 2022 [27], provided a long‐lived cellular model. After 1 week of culture, miR‐335‐5p was overexpressed in the organoids for another week. Immunofluorescence analysis was performed to assess the subcellular localization of thyroid‐specific proteins before and after 96 h of treatment. As shown in Fig. 7, both primary cell lines, particularly cell line 2, exhibited increased expression of all investigated thyroid‐specific markers (NIS, TPO, TSHR, and TG) following treatment with miR‐335‐5p. This increase was especially pronounced for TSHR and TG.

**Fig. 7:**
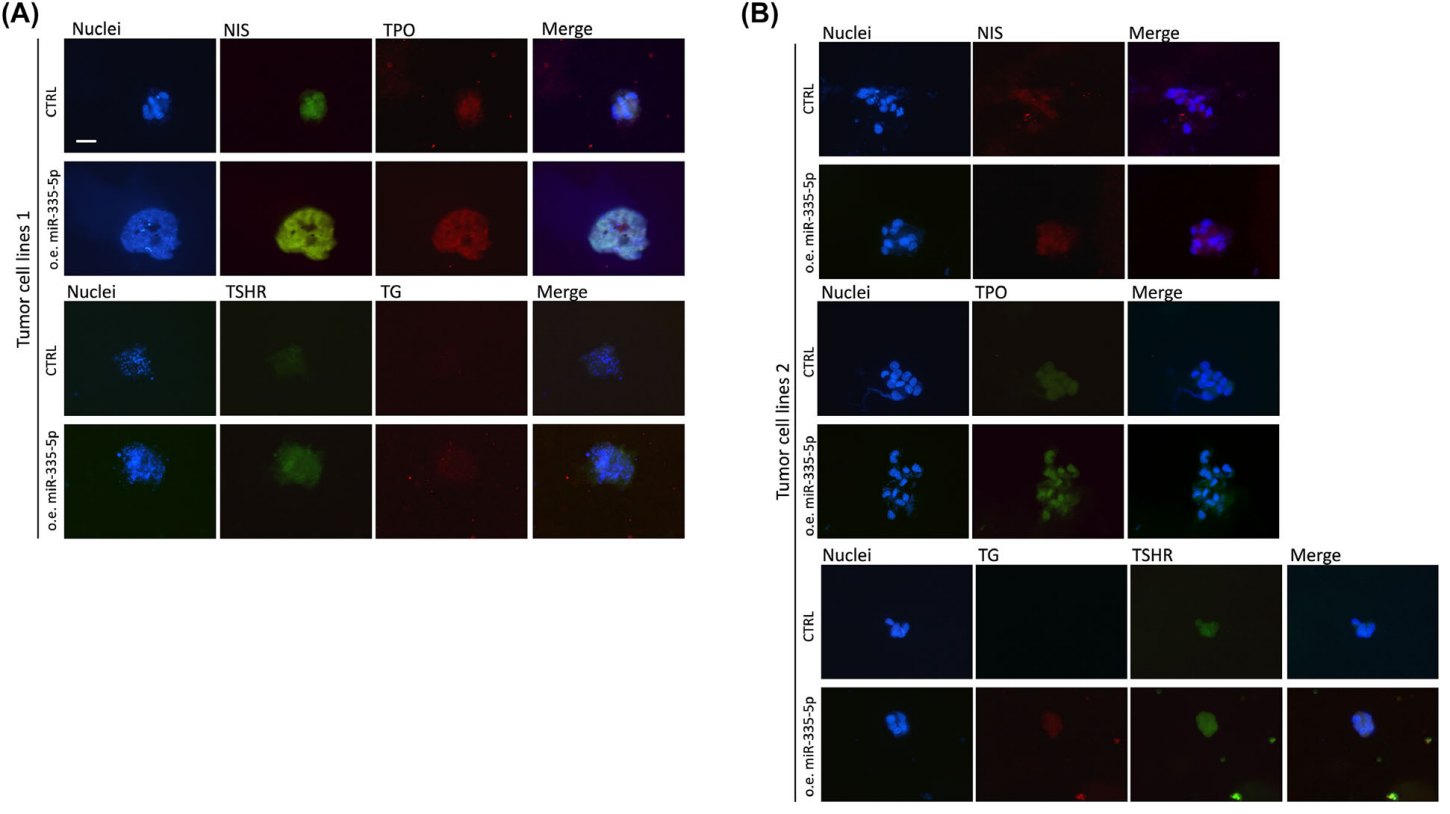
Thyroid‐specific protein localization in organoids. Immunofluorescence for localized NIS, TSHR, and TG on organoids generated with primary tumor cell line 1 (A) and 2 (B) before and after 96 h of miR‐335‐5p overexpression. The nuclei were stained with Hoechst (blue), NIS, TPO, TSHR, and TG in green or red. The images are represented at 40× magnification, with a scale bar of 100 μm. Representative immunofluorescence of three biologically independent replicates (*N* = 3).

These findings indicate that miR‐335‐5p overexpression influences the levels of thyroid‐specific proteins and promotes their membrane localization. Based on these results, we hypothesize that miR‐335‐5p is critical for maintaining thyroid‐specific protein levels, preserving thyroid‐specific polarization, and ensuring membrane localization of NIS.

To further validate this hypothesis, we evaluated NIS activity by analyzing iodine uptake after restoration of miR‐335‐5p in two primary and four immortalized cell lines. As shown in Fig. 8, intracellular iodine levels were significantly higher in miR‐335‐5p‐overexpressing cells compared to untreated cells, with statistically significant differences observed in all immortalized cell lines and primary cell line 1. In agreement with the protein result, the *BRAF*‐wt TPC1 cell line did not show significant changes in iodine uptake (Fig. S3A).

**Fig. 8:**
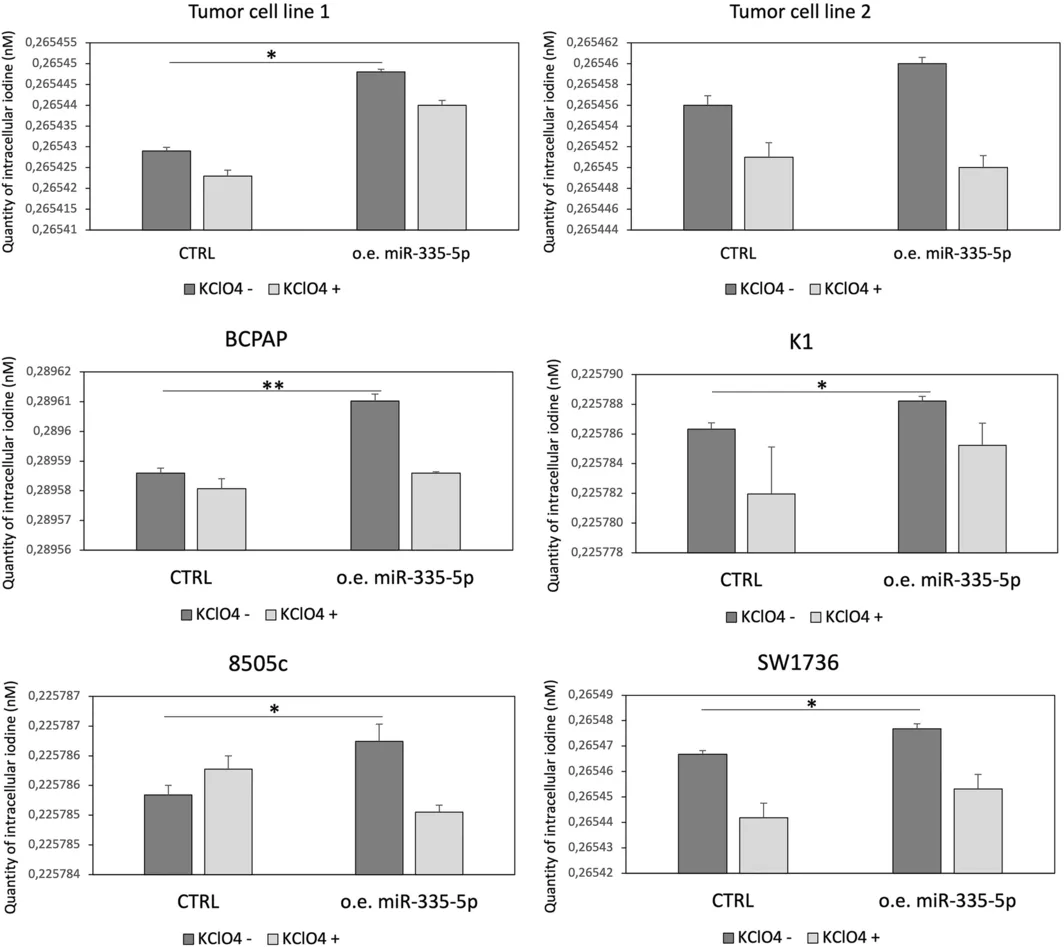
Iodine uptake in primary and immortalized cell lines. Intracellular iodine in primary tumor cell line 1, line 2, and immortalized cell lines (BCPAP, K1, 8505C, and SW1736) before and after the overexpression of miR‐335‐5p. The quantity of iodine is expressed as absolute concentration (nm) in cells with overexpressed miR‐335‐5p compared to control cells. The cells were treated or not with KClO_4_ 10 μm to control the NIS‐specific uptake. Data from three independent biological replicates (*N* = 3) are expressed as mean ± SD, *P* value < 0.05*; 0.005**; 0.0005*** (*t*‐test data in technical replicates).

Overall, these results suggest that miR‐335‐5p overexpression induces NIS post‐transcriptional modification, increasing its membrane localization and activation in all the *BRAF* p.V600E mutant cell lines regardless of their differentiation status.

### 3.5 The overexpression of miR‐335‐5p inhibits EMT‐related gene expression in poorly differentiated and anaplastic thyroid cancer cells

Previous experiments indicated that a stronger response to miR‐335‐5p treatment correlated with more differentiated cancer cells. To further explore this effect, we examined the influence of miR‐335‐5p overexpression on EMT‐related genes.

Analysis of the PPI network, constructed with the 227 switch genes, revealed two closely related clusters, including thyroid‐specific and EMT‐related genes, suggesting potential crosstalk between the dedifferentiation and EMT processes. Since miR‐335‐5p targets are present in both clusters, we investigated whether its overexpression could modulate genes of EMT‐related pathways that influence thyroid‐specific marker expression.

In four immortalized thyroid cancer cell lines, we evaluated the expression levels of genes involved in the TGF‐β (*FN1*, *MET*, *PDE5A*, *PLAUR*), Wnt/β‐catenin (*TCF3*), and Hippo (*LATS*, *YAP1*, *TAZ*) pathways, as well as downstream EMT markers (*CDH1*, *CDH16*, *CDH2*, *MMP9*), before and after miR‐335‐5p restoration. Almost all the selected genes are reported to interact with the thyroid‐specific genes in the PPI network built using the STRING tool (Fig. 9A).

**Fig. 9:**
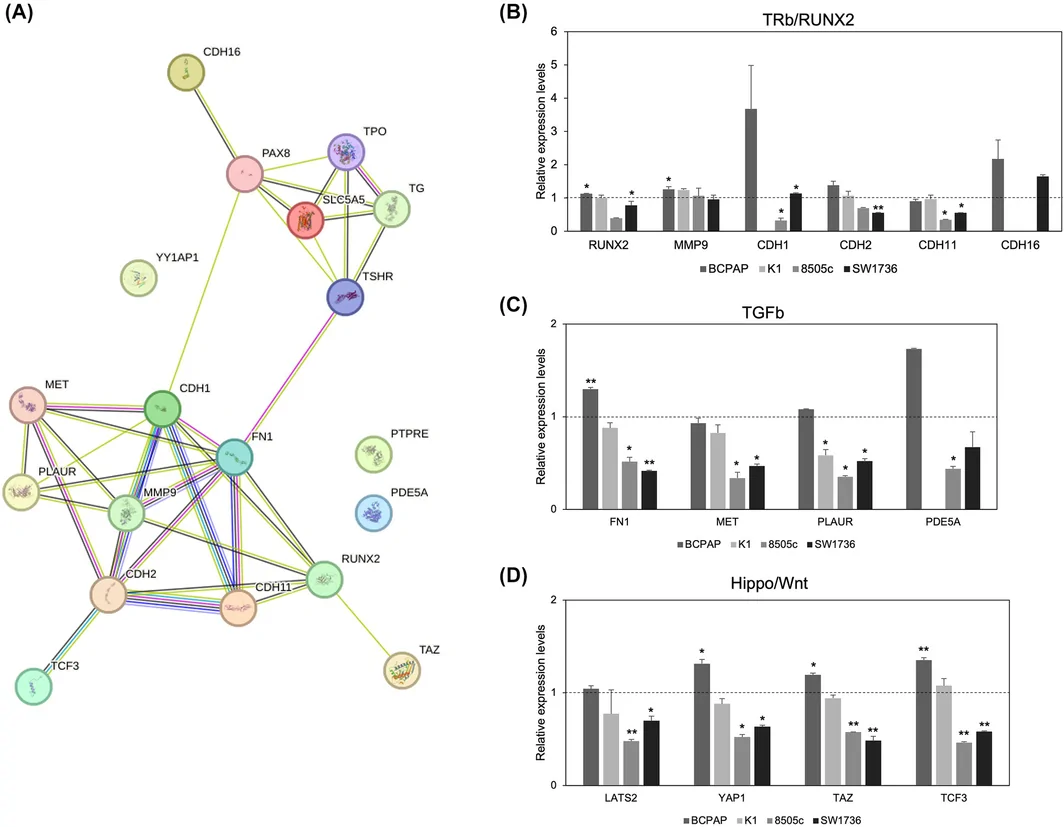
Epithelial to mesenchymal transition (EMT) in immortalized thyroid cancer cell lines. (A) Representation of protein–protein interaction (PPI) interactions among thyroid‐specific genes and epithelial‐mesenchymal transition (EMT)‐related gene clusters. Node colors indicate input proteins (blue), first‐shell interactors (green), and second‐shell interactors (yellow). Edge colors represent different sources of interaction evidence in STRING: green (gene fusion), red (co‐expression), blue (co‐occurrence), orange (gene neighborhood), purple (experimental evidence), light green (curated databases), and light blue (text mining). Relative expression levels of primary targets involved in EMT in immortalized cell lines BCPAP (B), K1 (C), 8505C (D), and SW1736 (E). The expression levels of EMT‐related genes are normalized to the endogenous control (B2M). Data from three independent biological replicates (*N* = 3) are presented as 2^−ΔΔCt^ values (mean ± SD) and compared with control cells (CTRL). *P* values < 0.05*, < 0.005**, < 0.0005*** (*t*‐test data).

As shown in Fig. 9B–D, the most dedifferentiated cell lines (8505c and SW1736) exhibited the most pronounced changes in EMT‐related gene expression, including *FN1*, *MET*, *PLAUR*, *CDH11*, *TCF3*, *LATS2*, *YAP1*, and *TAZ*. Notably, the anaplastic SW1736 cells also showed increased *CDH1* and *CDH16* expression. In contrast, the differentiated cell lines harboring the p.V600E mutation of *BRAF* exhibited minimal or no changes in gene expression. While the NTHYORI cell line showed almost no changes, the *BRAF*‐wt TPC1 cell line exhibited a reduction in several EMT‐related genes; however, these changes were not as pronounced as those observed in the dedifferentiated cell lines (Fig. S3B). To confirm the expression data, we performed an invasion assay. After restoring miR‐335‐5p, we monitored the invasive cells in each cell line harboring the p.V600E mutation of *BRAF* at 24 and 48 h. As shown in Fig. 10, the invasive capacity of all four cell lines was reduced at both 24 and 48 h, with the most significant effects observed in the less‐differentiated cell lines, 8505c and SW1736. The same assay was performed for the normal and *BRAF*‐wt control cell lines to determine whether this effect may directly depend on the *BRAF* p.V600E mutation. In all the control cell lines, we did not observe any change in invasive capability during the analyzed time point, including the TPC1 line (Fig. S1F).

**Fig. 10:**
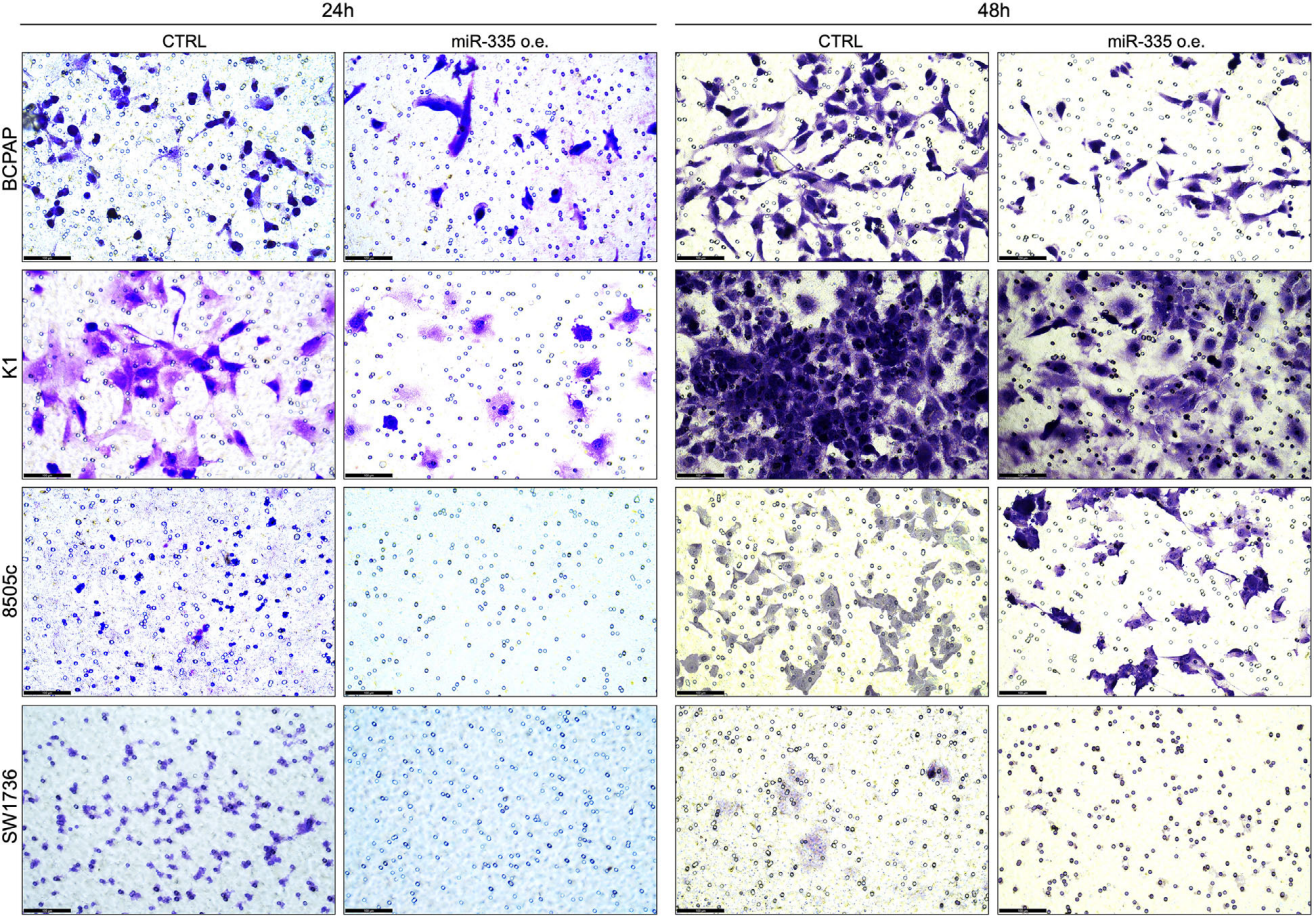
Invasion test before and after the restoration of miR‐335‐5p. Representative field of view of *BRAF*‐mutated invasive cell lines. Captured fields at 24 and 48 h after miR‐335‐5p overexpression. Cells were stained with crystal violet. The experiment was repeated in three independent replicates (*N* = 3). The scale bar corresponds to 100 μm.

## 4 Discussion

The redifferentiation strategy using BRAF and MEK inhibitors (BRAFi and MEKi) in metastatic radioactive iodine‐refractory (RAIR) differentiated thyroid carcinoma (DTC) has demonstrated effectiveness in restoring RAI uptake [10]. However, chronic, deleterious adverse effects, along with primary and acquired resistance to these inhibitors—partly due to the activation of compensatory mechanisms—limits the overall efficacy of this approach.

MicroRNAs (miRNAs), key regulators of cellular epigenetic processes, offer an alternative strategy for redifferentiating thyroid cancer.

Given the strong association between the *BRAF* p.V600E mutation and the loss of RAI avidity [4], we analyzed the previously identified regulatory switch genes involved in the *BRAF* p.V600E‐driven carcinogenesis of papillary thyroid carcinoma (PTC) to identify miRNAs that could facilitate redifferentiation. Our findings highlight miR‐335‐5p as a particularly promising candidate capable of regulating many switch genes.

Interestingly, we observed a progressive decrease in miR‐335‐5p expression from normal thyroid cells to benign lesions and malignant tumors, implicating its involvement in thyroid carcinogenesis. Although evidence on the role of miR‐335‐5p in differentiation has been provided for glioma, no data are available for thyroid cancer cells [31]. This observation led us to explore its potential role in promoting thyroid cancer redifferentiation. Overexpression of miR‐335‐5p restored the expression of nearly all thyroid‐specific genes and proteins in immortalized and well‐differentiated primary thyroid cancer cell lines but not in poorly differentiated ones, likely by an indirect mechanism. Notably, sodium‐iodide symporter (NIS) protein localization on the basolateral membrane was observed, facilitating functional intracellular iodide uptake even in less‐differentiated cell lines. This suggests miR‐335‐5p's involvement in NIS post‐transcriptional modifications in all thyroid cell lines regardless of their differentiation state.

However, miR‐335‐5p's redifferentiation effects appear to be *BRAF* p.V600E‐dependent. Indeed, BRAF‐wt control cell lines showed significant changes in NIS expression, localization, and iodine uptake. This is consistent with our initial network analysis, which focused on *BRAF* p.V600E‐mutant PTCs and excluded poorly differentiated and undifferentiated cancers. In the protein–protein interaction (PPI) network of switch genes, we identified two closely interconnected clusters: one comprising thyroid‐specific genes and the other containing genes involved in epithelial‐mesenchymal transition (EMT), suggesting a strong link between dedifferentiation and EMT [32]. We provided evidence that in the less‐differentiated thyroid cell lines, miR‐335‐5p overexpression modulated EMT‐associated pathways, including TGF‐β/Smad, Wnt/β‐catenin, and Hippo pathways, which are known to regulate thyroid‐specific gene expression [31]. These findings may be relevant since the investigated pathways are known to contribute to developing KI resistance in many cancers [33, 34, 35, 36, 37, 38].

Transforming growth factor β (TGF‐β), a potent EMT inducer, plays a role in normal thyroid cell differentiation. It notably inhibits PAX8 binding to the *NIS* enhancer region, thereby decreasing *NIS* expression [39]. The miR‐335‐5p overexpression downregulated key genes of the TGF‐β pathway participating in the EMT process, such as *FN1*, *MET*, *PLAUR*, and *PDE5A*. Previous studies showed that *PDE5A* and *FN1* were overexpressed in *BRAF*‐mutant thyroid cancer tissues, and their inhibition reduced proliferation and migration in *BRAF*‐mutant thyroid cancer cells [40, 41], supporting miR‐335‐5p's potential therapeutic value. Notably, *MET* amplification and *PLAUR* overexpression have been found involved in the acquired resistance to BRAFi [42, 43].

The Wnt/β‐catenin pathway regulates thyroid cell differentiation. At the physiological level, β‐catenin interacts with PAX8 to enhance NIS transcription, while aberrant β‐catenin expression in the nucleus is associated with a dedifferentiation effect [10]. β‐catenin also interacts with TCF3, a transcriptional repressor, regulating several processes, including EMT [44]. As reported in the hTFtarget database (https://guolab.wchscu.cn/hTFtarget/#!/), among TCF3's targets are *PAX8*, *TSHR*, and *TG*. Overexpression of miR‐335‐5p reduced aberrant β‐catenin expression, promoting thyroid cell differentiation.

Similarly, miR‐335‐5p inhibited the Hippo pathway, decreasing *LATS2*, *YAP1*, and *TAZ* expression, with *TAZ* known to enhance NIS expression, membrane localization, and function [45]. Moreover, miR‐335‐5p decreased the expression levels of *RUNX2*, an EMT‐associated transcription factor. *RUNX2* is repressed by the thyroid hormone receptor β (TRβ), and the TRβ/RUNX2 ratio appears to correlate with the differentiation grade of thyroid cancers [46]. The modulation of all investigated pathways by miR‐335‐5p led to a decrease in mesenchymal markers (e.g., *CDH2*, *CDH11*, and *MMP9*) and an increase in epithelial markers (e.g., *CDH1* and *CDH16*), further demonstrating miR‐335‐5p's impact on EMT in thyroid cancer, as previously shown in other cancers, resulting in the reduction of invasive capacity [47, 48, 49]. For some of the genes investigated, the effect of miR‐335‐5p may be direct. Indeed, *MMP9*, *PDE5A*, *LATS*, *TAZ*, *YAP1*, and *RUNX2* are reported as validated direct targets of miR‐335‐5p in miRTarBase. Our findings provide evidence for miR‐335‐5p as a possible key tumor suppressor in thyroid cancer, essential for maintaining thyrocyte differentiation. Its reduction in *BRAF* p.V600E‐mutated thyroid cancers likely contributes to dedifferentiation by impairing thyroid‐specific markers at multiple levels, suggesting the involvement of an indirect mechanism. As dedifferentiation progresses, miR‐335‐5p's regulatory effects diminish, enabling the upregulation of pathways like TGF‐β/Smad, Wnt/β‐catenin, and Hippo, which promote EMT and cancer progression.

## 5 Conclusion

Our results are consistent with previous studies associating miR‐335‐5p with reduced cancer growth and aggressiveness in well‐differentiated and anaplastic thyroid cancers [50, 51].

In conclusion, miR‐335‐5p represents a potential therapeutic target to restore RAI avidity in *BRAF* p.V600E‐mutant metastatic RAIR DTC. Due to its inhibitory effect on pathways involved in thyrocyte differentiation, we can speculate that it may be used in combination with BRAFi and MEKi to enhance treatment efficacy. Moreover, it may be used to potentiate the effect of other miRNAs, such as miR‐139‐5p, whose differentiated role in RAIR DTC has been previously demonstrated [12].

Further research, including in‐depth analysis of the involved molecular mechanisms and *in vivo* validation, is warranted to evaluate miR‐335‐5p's potential in clinical settings and its role in reactivating RAI uptake in refractory thyroid cancers.

## Conflict of interest

The authors declare no conflict of interest.

## Author contributions

VP, SB, MS, and VM performed the experiments and analyzed the data; GG and RF contributed to data interpretation; GF, PP, and LF performed the *in silico* analysis and the data interpretation; SF, CD, and AV conceptualized and designed the study. All authors contributed to writing, reviewing, and approving the final manuscript.

## Supporting information


**Fig. S1.** Expression and function of thyroid‐specific genes in the control cell line. (A) Basal expression of miR‐335‐5p in TPC1 and FTC‐133 cancer cell lines compared with NTHYORI normal cell line. (B) Expression levels of miR‐335‐5p in NTHYORI, TPC1, and FTC‐133 cell lines after 48 h of miRNA overexpression. (C) Expression analysis of *TSHR*, *PAX8*, *TG*, *TPO*, and *NIS*, before and after miR‐335‐5p transfection. (D) Representative western blot of TPO, TSHR, NIS, and PAX8, and (E) densitometric analysis of two biological replicates in NTHYORI, TPC1, and FTC‐133 cell lines before and after the overexpression of miR‐335‐5p. (F) Representative field of view (1 out of 3 for each condition) of the invasion assay, cells were stained with crystal violet.

**Fig. S2.** miR‐335‐5p restoration in thyroid cell lines. Expression levels of miR‐335‐5p after 48 h of transfection in primary cell lines (A) and immortalized cell lines (B). Data are expressed as mean ± SD, normalized to the endogenous control (snRNA U6), and compared with control (CTRL) cells, *P* value < 0.05, *; 0.005 **; 0.0005 *** (*t*‐test data).

**Fig. S3.** EMT and iodine uptake in control cell lines. (A) Intracellular iodine levels TPC1 before and after miR‐335‐5p overexpression. Iodine concentration is expressed as absolute values (nm) in cells overexpressing miR‐335‐5p compared with control cells. Cells were treated or not with KClO₄ (10 μm) to assess NIS‐specific uptake. Statistical significance: *P* < 0.05 (*), < 0.005 (**), < 0.0005 (***) (*t*‐test). (B) Expression levels of EMT targets in normal thyroid cells (NTHYORI) and the BRAF‐wt cell line TPC1. Data represent expression levels after treatment with miR‐335‐5p for 48 h; the dotted line indicates the levels in untreated controls. Results are expressed as mean ± SD and normalized to the endogenous control (Actin).

**Table S1.** Switch genes in thyroid cancer with p.V600E mutation of *BRAF* gene.

**Table S2.** Mienturnet Enrichment results miRTarBase.

## Data Availability

Additional data and materials supporting this study are provided in the Supporting Information. Further methodological details or clarifications can be made available by the authors upon request.
