# Supplementary figures and images for: The role of miR‐335‐5p in the redifferentiation of BRAF p.V600E thyroid cancers
Source: Mol Oncol. 2026 Jun 22:10.1002/1878-0261.70181. Online ahead of print. doi: 10.1002/1878-0261.70181 (PMC13398929; doi:10.1002/1878-0261.70181)

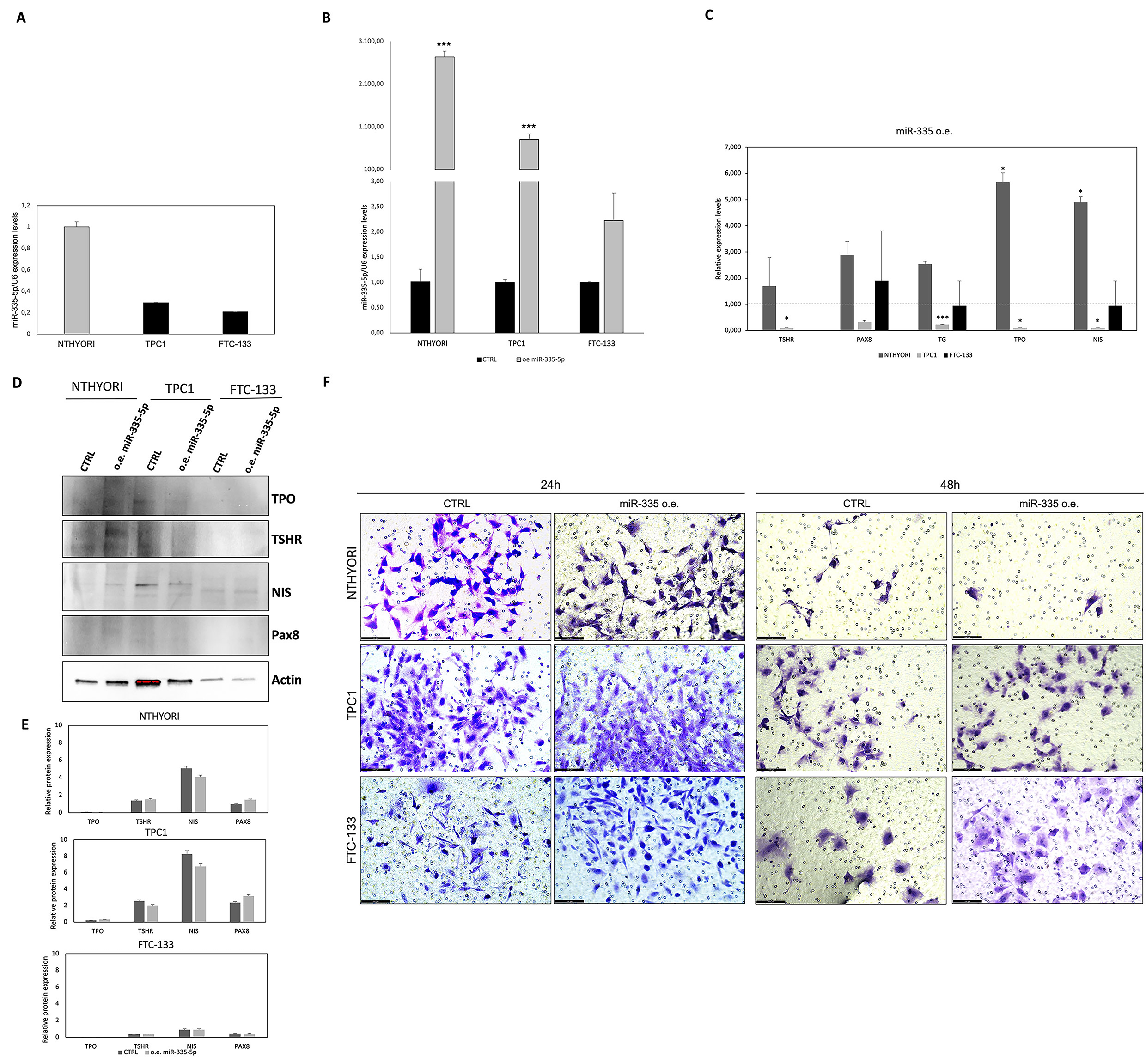

Supplement: Supplementary file 1 — Fig. S1. Expression and function of thyroid‐specific genes in the control cell line. (A) Basal expression of miR‐335‐5p in TPC1 and FTC‐133 cancer cell lines compared with NTHYORI normal cell line. (B) Expression levels of miR‐335‐5p in NTHYORI, TPC1, and FTC‐133 cell lines after 48 h of miRNA overexpression. (C) Expression analysis of TSHR, PAX8, TG, TPO, and NIS, before and after miR‐335‐5p transfection. (D) Representative western blot of TPO, TSHR, NIS, and PAX8, and (E) densitometric analysis of two biological replicates in NTHYORI, TPC1, and FTC‐133 cell lines before and after the overexpression of miR‐335‐5p. (F) Representative field of view (1 out of 3 for each condition) of the invasion assay, cells were stained with crystal violet. [file MOL2-9999-0-s002.tif]

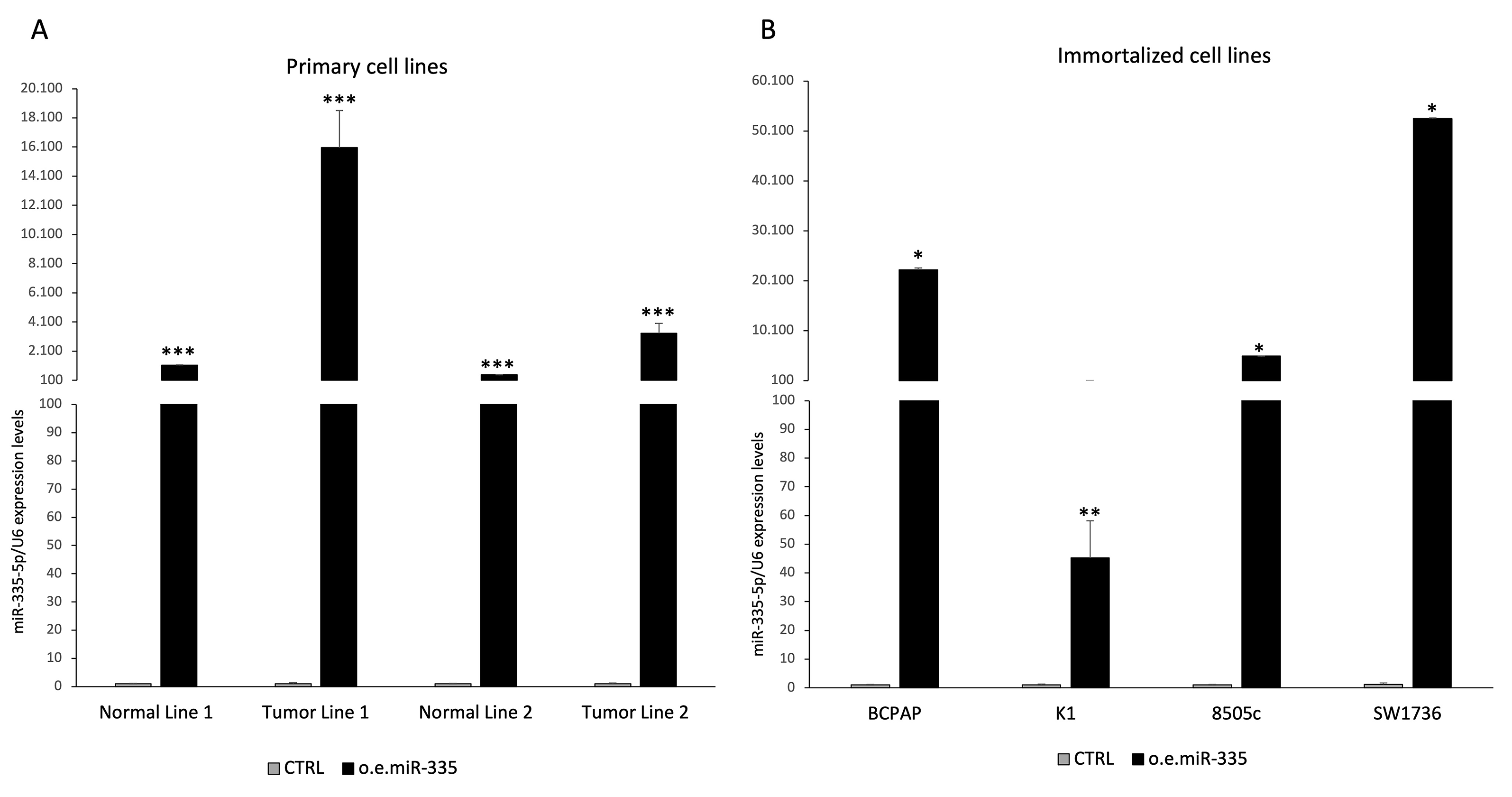

Supplement: Supplementary file 2 — Fig. S2. miR‐335‐5p restoration in thyroid cell lines. Expression levels of miR‐335‐5p after 48 h of transfection in primary cell lines (A) and immortalized cell lines (B). Data are expressed as mean ± SD, normalized to the endogenous control (snRNA U6), and compared with control (CTRL) cells, P value < 0.05, *; 0.005 **; 0.0005 *** (t‐test data). [file MOL2-9999-0-s001.tif]

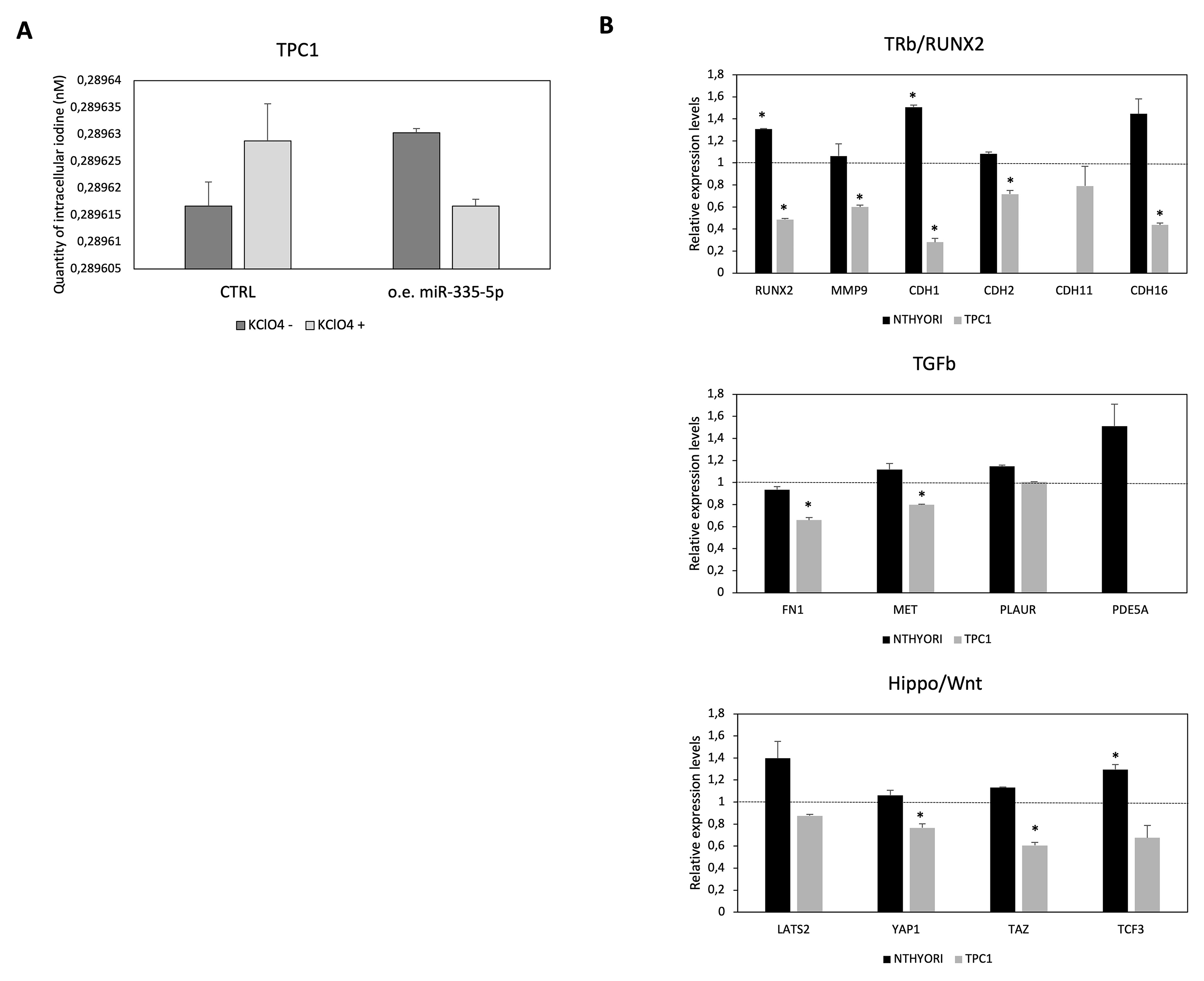

Supplement: Supplementary file 3 — Fig. S3. EMT and iodine uptake in control cell lines. (A) Intracellular iodine levels TPC1 before and after miR‐335‐5p overexpression. Iodine concentration is expressed as absolute values (nm) in cells overexpressing miR‐335‐5p compared with control cells. Cells were treated or not with KClO₄ (10 μm) to assess NIS‐specific uptake. Statistical significance: P < 0.05 (*), < 0.005 (**), < 0.0005 (***) (t‐test). (B) Expression levels of EMT targets in normal thyroid cells (NTHYORI) and the BRAF‐wt cell line TPC1. Data represent expression levels after treatment with miR‐335‐5p for 48 h; the dotted line indicates the levels in untreated controls. Results are expressed as mean ± SD and normalized to the endogenous control (Actin). [file MOL2-9999-0-s004.tif]
